# Bavachin ameliorates cisplatin-induced nephrotoxicity by enhancing mitochondrial β-oxidation and lipid metabolism through MFN2

**DOI:** 10.1186/s10020-025-01283-6

**Published:** 2025-06-11

**Authors:** Shilu Luo, Ming Yang, Na Jiang, Chenrui Li, Yan Liu, Lin Sun

**Affiliations:** 1 Department of Nephrology, the Second Xiangya Hospital, Central South University, No. 139 Renmin Middle Road, Changsha, Hunan 410011 China; 2 Hunan Key Laboratory of Kidney Disease and Blood Purification, No. 139 Renmin Middle Road, Changsha, Hunan 410011 China

**Keywords:** Bavachin, Acute kidney injury, MFN2, Oxidative stress, Lipid accumulation, Mitochondrial dynamics, Renal protection

## Abstract

**Background:**

Cisplatin-induced nephrotoxicity is a critical adverse reaction that restricts the clinical utilization of cisplatin. Alterations in fatty acid metabolism have been associated with the pathogenesis of cisplatin-induced nephrotoxicity, yet the precise mechanisms remain unclear. Bavachin, a natural flavonoid, exhibits anti-inflammatory, antioxidant, and lipid metabolism-regulating properties, yet its role in mitigating cisplatin-induced nephrotoxicity via mitochondrial β-oxidation remains unexplored. Mitofusin-2 (MFN2), a mitochondrial fusion protein, has emerged as a critical regulator of fatty acid oxidation (FAO) and lipid homeostasis. However, its role in cisplatin-induced nephrotoxicity has not been fully explored.

**Methods:**

C57/6L mice were randomly divided into control, DMSO, cisplatin, and cisplatin + Bavachin groups. Blood urea nitrogen (BUN), serum creatinine (SCr), reactive-oxygen-species (ROS), lipid accumulation, and apoptosis were assessed. In vitro, the human proximal tubule epithelial cell line (HK-2) cells were treated with 20 µM cisplatin with or without bavachin. ROS production was detected by the DCFH-DA, lipid deposition was detected by oil red O staining, and MFN2, carnitine palmitoyltransferase 1a (CPT1a) were detected by Western blot (WB).

**Results:**

Compared with the cisplatin group, bavachin treatment reduced BUN (21.8%) and SCr (78.7%) in the cisplatin group, accompanied by improvements in renal pathological changes, lipid deposition, and apoptosis. In addition, bavachin up-regulated the expression of MFN2 and CPT1a, while decreasing the cisplatin-induced ROS overproduction. Similar results were found in vitro. Notably, the mitochondrial FAO has been increased in HK-2 cells treated with bavachin. Further, MFN2 siRNA partially reversed these protective effects, accompanied by decreased CPT1a expression and exacerbated lipid deposition.

**Conclusions:**

This study is the first to confirm MFN2 as a target for renal protection by bavachin. Mechanistically, Bavachin alleviated cisplatin-induced lipid accumulation and apoptosis by upregulating MFN2 expression, which activated CPT1a to promote mitochondrial FAO. These results will provide a new strategy for cisplatin-based cancer therapy and the reduction of its nephrotoxicity.

**Supplementary Information:**

The online version contains supplementary material available at 10.1186/s10020-025-01283-6.

## Introduction

Acute kidney injury (AKI) developed in approximately 30% of adult cancer patients following the administration of a singular dose of cisplatin, despite its cost-effectiveness and notable effectiveness in combating solid tumors (Latcha, et al. 2016). Recent studies have shown that individuals who receive intravenous cisplatin are at the highest risk of AKI (Gupta, et al. 2024). The uptake of cisplatin within renal tubular cells surpasses plasma levels by a factor of five, rendering these cells particularly susceptible to cisplatin-induced toxicity. The metabolic transformation of cisplatin within renal tubular cells leads to the generation of more potent metabolites, triggering oxidative stress, inflammation, apoptosis, and necrosis (Curry and McCormick 2022). However, the precise mechanism basis remains elusive, and effective renal protection strategies are currently lacking.

Fatty acid metabolism serves as the primary energy source for renal tubular cells, and the imbalance of FAO is involved in the transition from AKI to chronic kidney disease (CKD) (Simon and Hertig 2015; Xu, et al. 2023) and promotes the onset and progression of AKI through various mechanisms such as lipotoxicity, inflammation, and oxidative stress. Lipidomics revealed significant changes in kidney lipid discovery induced by cisplatin during AKI (Moreno-Gordaliza, et al. 2021). In addition, metabolomic analysis of kidney and urine samples of cisplatin-induced AKI mice revealed seven key metabolic pathways involved in energy production, and disruption of amino acid and lipid metabolism, which may lead to oxidative stress damage, inflammation, and cell membrane damage (Qu, et al. 2020). Impaired fatty acid oxidation and lipid accumulation are important features of cisplatin-induced AKI, and many studies have found that enhanced fatty acid oxidation can improve AKI (Sun, et al. 2022; Wang, et al. 2024; Xiong, et al. 2021; Xu, et al. 2022). In addition, Xiong et al. shown that cisplatin induced AKI was associated with significant lipid accumulation and was positively correlated with renal injury (Xiong, et al. 2021). The mechanism may be related to the direct inhibition of peroxisome proliferator-activated receptor gamma coactivator-1 α(PGC-1α) by cisplatin, which leads to a decrease in the activity of CPT1a and peroxisome proliferator-activated receptor α (PPARα), and hinders FAO (Wang, et al. 2024). Other studies have also found that in cisplatin induced AKI, upregulation of UCP1 can activate AMPK/ULK1/autophagy pathway, reducing lipid accumulation and kidney injury (Xiong, et al. 2021). Furthermore, farnesoid X receptor (FXR) improves cisplatin-induced lipid accumulation, tubular damage, and decreased renal function by activating PPARγ to promote FAO (Xu, et al. 2022). However, the role of bavachin in the regulation of FAO in AKI remains unclear.

Bavachin, a natural flavonoid with bioactive properties (Li, et al. 2020), exhibits antioxidant, anti-apoptotic, and anti-inflammatory effects (Alam, et al. 2018). In Ban et al., bavachin was found to inhibit oxidative stress and inflammation, thereby improving LPS-induced AKI (Ban, et al. 2022). Bavachin has shown the potential to ameliorate diabetic nephropathy by upregulating antioxidant enzymes and mitochondrial function factors in renal tissues (Park, et al. 2023). Additionally, previous studies have suggested that bavachin may play a role in regulating lipid metabolism. In hepatocyte, bavachin significantly reduced the expression of genes associated with fatty acid synthesis, including sterol regulatory element binding protein 1c, PPARγ, stearoyl-CoA desaturase 1 and fatty acid synthase, and increased phosphorylation levels of AKT and glycogen synthase kinase 3β (Wei, et al. 2023). This suggests that bavachin has a regulatory effect on hepatic lipid metabolism, but the study on renal lipid metabolism in AKI state needs to be further confirmed. A recent study by Abass et al. found that papaverine, an alkaloid derived from *Papaver somniferum*, inhibits oxidative stress, inflammation, and apoptosis in a cisplatin-induced kidney injury model via the MAPK1 pathway, while not affecting the anticancer activity of cisplatin (Abass, et al. 2024). However, there is still a gap in targeted drugs for kidney injury caused by lipid deposition. Bavachin has been shown to be protective against LPS-induced AKI (Ban, et al. 2022) and has properties that regulate lipid metabolism (Wei, et al. 2023), potentially preventing subsequent chronic damage such as fibrosis. However, whether bavachin regulates lipid metabolism and protects against cisplatin-induced AKI remains to be investigated.

Mitofusin 2 (MFN2), a fusion protein located in the outer membrane of mitochondria, is not only directly involved in mitochondrial dynamics, but also affects cellular energy balance by regulating fatty acid oxidative metabolism pathways (Schrepfer and Scorrano 2016). MFN2 governs mitochondrial architecture and bioenergetic capacity, where its deficiency triggers ROS production and compromised OXPHOS efficiency, while simultaneously orchestrating inter-organellar lipid homeostasis, with loss-of-function impairing ER-mitochondrial phosphatidylserine trafficking(Hernández-Alvarez, et al. 2019; Naón, et al. 2023). MFN2 is involved in various diseases such as pulmonary fibrosis (Chung, et al. 2019), nonalcoholic fatty liver disease (NAFLD) (Bórquez, et al. 2024), and myocardial lipotoxicity (Hu, et al. 2024). Notably, a recent research indicated that MFN2 not only inhibits mitochondrial fragmentation, proliferation, migration, and invasion in clear cell renal cell carcinoma (ccRCC) cells and xenograft tumor growth but also suppresses disease progression and improves prognosis in ccRCC patients by blocking cellular lipid metabolism and reducing lipid droplet accumulation (Cai, et al. 2024). Additionally, the expression level of MFN2 was significantly decreased in retinal ischemia/reperfusion injury (IRI)-induced AKI, leading to increased mitochondrial fragmentation and apoptosis. Furthermore, the expression of MFN2 in liver was also decreased, and Treprostinil significantly upregulated MFN2 level by restoring PGC-1α expression, improving linear liver particle dynamics (Hou, et al. 2021). MFN2 expression decreased in cisplatin-induced podocytes (Gong, et al. 2023). High fat intervention can reduce MFN2 expression, increase mitochondrial fragmentation, and impair mitochondrial energy metabolism (Ding, et al. 2022). These results suggest that MFN2 regulates mitochondrial dynamics and lipid metabolism. However, whether MFN2 regulates lipid metabolism in cisplatin-induced AKI has not been reported. We hypothesized that Bavachin protects against cisplatin-induced AKI via MFN2-mediated regulation of mitochondrial β-oxidation and lipid metabolism. Due to the high biocompatibility and low toxicity of bavachin, the purpose of this study is to provide a new therapeutic strategy for the future clinical use of bavachin as cisplatin in the treatment of tumor-related diseases.

## Materials and methods

### Animals model

6-to-8-week male C57BL/6 mice (body weight range: 21–25 g) purchased from Hunan SJA Laboratory Animal Co.Ltd. All mice were placed in a quiet environment (temperature: 21–24 °C; Humidity: 50 ± 5%) and had free access to water and standard chow. All experimental mice were randomly divided into control, DMSO, cisplatin, and cisplatin + Bavachin group. The male C57BL/6 mice in the cisplatin group were injected with cisplatin (20 mg/kg, cat # 479306, Sigma, United States) intraperitoneally and the control group was given the same volume of saline. Mice in the cisplatin + Bavachin group were administered Bavachin (20 mg/kg) by single intraperitoneal injection, as previously described (Ban, et al. 2022; Wei, et al. 2023). Bavachin (MedChemExpress, HY-N0233) was dissolved in 10% dimethyl sulfoxide and 90% saline mixed with sulfobutylether-β-cyclodextrin (MedChemExpress, HY-17031). All mice were euthanized with pentobarbital at 72 h. All animal experiments were performed by the protocols approved by the Institutional Animal Care and Use Committee of the Second Xiangya Hospital of Central South University.

### Serum biochemistry

After centrifugation at 3000 rpm for 15 min, the obtained serum was used to detect blood urea nitrogen, serum creatinine (SCr), and serum cholesterol (CHOL). The commercial assay kits for the above tests were obtained from FUJIFILM Wako Pure Chemical Corporation, CicaLiquid-N CRE (KANTO Chemical CO., Inc), and Medicalsystem Biotechnology CO., Ltd.

### Renal histology

The kidneys of all mice were fixed with 4% paraformaldehyde, dehydrated, and then cut into 5-μm thick sections. The score of kidney damage was evaluated by hematoxylin and eosin staining (H&E). The renal tissue damage score from 0 to 4 was given for pathological assessment: 0, normal histology; 1, mild in jury, 5% to 25%of tubules showed pathological damage; 2, moderate injury, 25% to 50% of tubules showed pathological damage; 3, severe injury, 50% to 75% showed pathological damage; and 4, more than 75% damage (Basile, et al. 2012; Xu, et al. 2022). The images were captured with a microscopy (Leica DMI3000 B, Germany).

### Western blot

The renal cortex tissues were lysed in RIPA Buffer + proteinase inhibitor + phosphatase inhibitor. Total protein extracts were subjected to electrophoresis in SDS-PAGE gels and blotted onto PVDF membranes. The membranes were blocked with 5% fat-free milk for 2 h and incubated with primary antibodies at 4 °C overnight. The primary antibodies included: KIM-1/TIM-1 (1:1000, sc-518008, Santa cruz biotechnology); NGAL (1:5000, AF1857, R&D systems); CPT1α (1:5000, 15184–1-AP, Proteintech); MFN2 (1:5000, 12186–1-AP, Proteintech); BAX (1:5000, 60267–1-Ig, Proteintech); BCL-2(1:1000, af6139, Affinity); β-actin (1:10000, 60008–1-Ig, Proteintech). Then the membranes were incubated with the HRP-conjugated Goat Anti-Rabbit IgG(H + L) (1:5000, SA00001-2, Proteintech) or HRP-conjugated Goat Anti-Mouse IgG(H + L) (1:5000, SA00001-1, Proteintech). After washing, images were detected by Tanon-5200Multi instrument (Tanon Instruments). Quantification of the band intensity were measured by ImageJ software (National Institutes of Health).

### Immunohistochemical

Sections of kidney tissue 5 μm thick were deparaffinization, dehydration, antigen retrieval, and 10-min incubation in 3% H2O2, incubated in 5% BSA for 1 h, washed 3 times with PBS, and incubated with KIM-1/TIM-1(1:250, sc-518008, Santa Cruz biotechnology), ADRP (1:250, 15294–1-AP, Proteintech), and MFN2 (1:250, 12186–1-AP, Proteintech) at 4℃ overnight. Subsequently, they were incubated with immunohistochemical specific goat anti-rabbit (1:200, G1213-100UL, Servicebio) or anti- mouse secondary antibody (1:200, G1214-100UL, Servicebio) at room temperature, treated with diaminobenzidine, stained with hematoxylin, dehydrated with alcohol gradient, air dried, sealed with neutral resin, and observed under a light microscope. The staining intensity of KIM-1, ADRP, and MFN2 was quantified using ImageJ software (National Institutes of Health).

### Immunofluorescent staining

The kidney tissue paraffin sections (5 μm) were deparaffinized and dehydrated, followed by antigen retrieval and 1-h incubation in 5% BSA. Subsequently, the sections were incubated overnight at 4 °C with ADRP (1:250, 15294–1-AP, Proteintech) and CPT1α (1:300, 15184–1-AP, Proteintech) antibodies. After PBS washing, the sections were incubated with Alexa Fluor-488-labeled secondary antibodies (1:1000, ab150077, Abcam), and the cell nuclei were stained with 4,6-diamidino-2-phenylindole (DAPI). Fluorescence intensity of ADRP and CPT1α was quantified using ImageJ software (National Institutes of Health).

### Cell culture

HK-2 cells, a proximal tubular cell (PTC) line, were procured from the American Type Culture Collection, and were incubated in DMEM/F12 medium, 10% fetal bovine serum, and 1% ampicillin-streptomycin solution. HK-2 cells were cultured in a 37 °C, 5% CO2 environment. When the cells reached 70–80% confluency, the cisplatin group was treated with 20 µM cisplatin alone, while the cisplatin + Bavachin group was treated with 20 µM cisplatin plus 5/10/20 mM Bavachin for 24 h. For gene silencing, MFN2 siRNA was transfected into HK-2 cells using a Lipofectamine 3000 reagent (Invitrogen) before cisplatin treatment, followed by overnight culture. The morphological changes and apoptosis in HK-2 cells were observed by staining with Hoechst 33342. Prior to the experiment, HK-2 cells were identified to confirm their identity and were tested for contamination by mycoplasma reagents (Biosharp, BL1469 A).

### Analysis of reactive oxygen species (ROS) production

Dihydroethidium (DHE) and DCFH-DA (Beyotime) staining were used to detect ROS production in kidney tissues and HK-2 cells, respectively. Fresh frozen kidney sections were incubated with DHE (Invitrogen) probe (1 μM working solution) at 37℃ for 30 min. Fluorescence signals were captured using a fluorescence microscope (Leica DMI3000 B, Germany) with an excitation wavelength of 535 nm and emission wavelength of 610 nm (red channel). DCFH-DA was diluted with serum-free culture medium at 1:1000, the cell culture medium was removed, and the diluted DCFH-DA was added in an appropriate volume and incubated in a cell incubator at 37℃ for 20 min. Fluorescence intensity was quantified using a microplate reader (Leica DMI3000 B, Germany) with excitation/emission wavelengths set to 488 nm/525 nm (green channel).

### Oil Red O staining

Fresh frozen kidney tissue sections (5 μm) were dried for 1 h and cleaned 3 times with PBS, then treated with 60% isopropyl alcohol for 20–30 s and stained with oil red O working liquid (G1015, Servicebio) (supernatant: distilled water = 3:2) for 10 min. After washing with PBS, hematoxylin was used for nucleation.

HK-2 cells were washed with PBS, fixed in 4% paraformaldehyde for 5 min, differentiated by 60% isopropyl alcohol for 20–30 s, stained with oil red O for 10 min, and then stained with hematoxylin after washing with PBS. Image Pro plus 6.0 (Media Cybernetics) was used to analyze the oil red O staining results and calculate the area percentage.

### Molecular docking

The files of sdf. structure of Bavachin was downloaded from the PubChem database, and imported into Open Babel-3.1.1 software to save in PDB format. After AutoDockTools 1.5.6 processing, the files were saved in pdbqt. format. The three-dimensional crystal structure of MFN2 was downloaded from the PDB protein database (https://www.rcsb.org/) and the PDBIDs was MFN2 (6jfm). The water molecule and organic matter in the target protein were removed by Notedad2 software, and then the target protein was imported into AutoDockTools1.5.6 for hydrogenation, charge distribution, and atomic type addition. The pdbqt. format file was saved. Molecular docking was performed using AutoDockVina, and the docking results were displayed using PyMOL (Gu, et al. 2024).

### Statistical analysis

The assumptions of normality and homoscedasticity were verified prior to analysis. All qualitative data were expressed as mean ± SD. The differences between 2 groups were analyzed using Student’s *t*-test, while those between multiple groups (≥ 3) were analyzed using one-way ANOVA analysis of variance, followed by Tukey’s post-hoc test. *p* < 0.05 was considered significant. All statistical analyses in this study were performed using Prism version 8.0.2.

## Results

### Bavachin ameliorates renal tubular injury in cisplatin-induced AKI mice

**Fig. 1:**
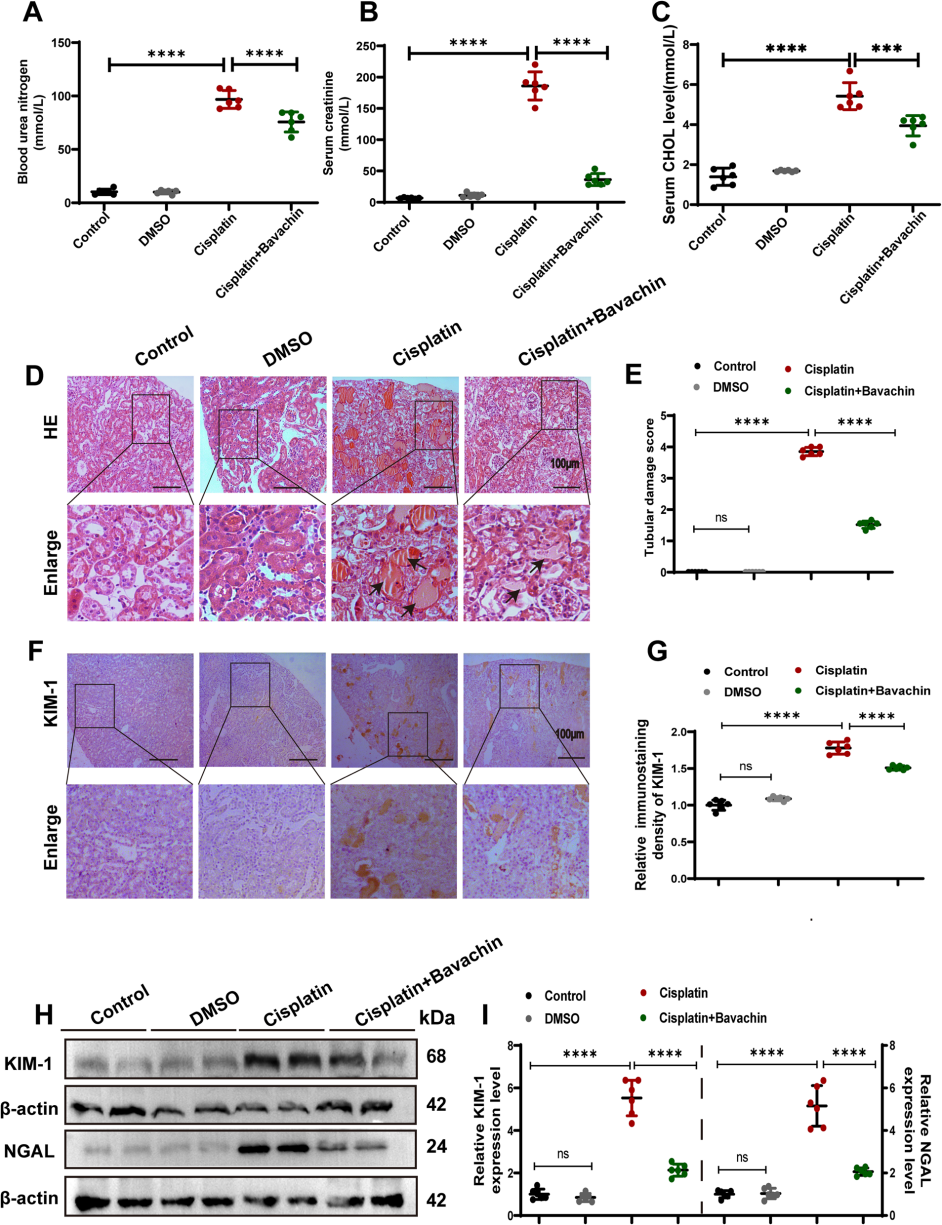
Bavachin ameliorates renal tubular injury in cisplatin-induced AKI mice. **A**, **B** serum creatinine and blood urea nitrogen levels after 72 h of cisplatin administration.** C** Serum cholesterol levels in each group of mice. **D** Representative images of hematoxylin and eosin staining. Scale bar = 100 μm. **E** Tubular damage score. **F** Representative image of immunohistochemical staining of KIM-1 in renal tissues from each group. Scale bar = 100 μm. ** G** Relative immunostaining density of KIM-1. **H** Representative western blotting analysis of KIM-1 and NGAL protein levels in mouse kidneys.** I** Relative band intensity of KIM-1 and NGAL. *n* = 6 animals per group. All data were expressed as the mean ± SD (ns, no significant. ****p < 0.0001)

Compared with the control group, the SCr, BUN, and serum cholesterol were significantly increased in cisplatin-induced AKI mice, while significantly decreased after bavachin treatment (Fig. 1A-C). H&E staining revealed severe tubular necrosis and protein casting formation in the kidney of cisplatin-induced AKI mice, which were ameliorated following treatment with bavachin (Fig. 1D, E). In addition, immunohistochemistry staining of KIM-1, a marker of renal tubular cell injury, showed that KIM-1 was significantly upregulated in cisplatin-induced renal tissue, while was significantly down-regulated in cisplatin + Bavachin group (Fig. 1F, G). Furthermore, WB analysis revealed a significant increase in the protein expression levels of KIM-1 and NGAL in cisplatin-induced AKI mice compared to the control group, while a notable reduction was observed in the bavachin intervention group (Fig. 1H, I). This data indicates that bavachin has a protective effect on cisplatin-induced AKI.

### Bavachin attenuates cisplatin-induced renal oxidative stress, lipid accumulation, and cell apoptosis

**Fig. 2:**
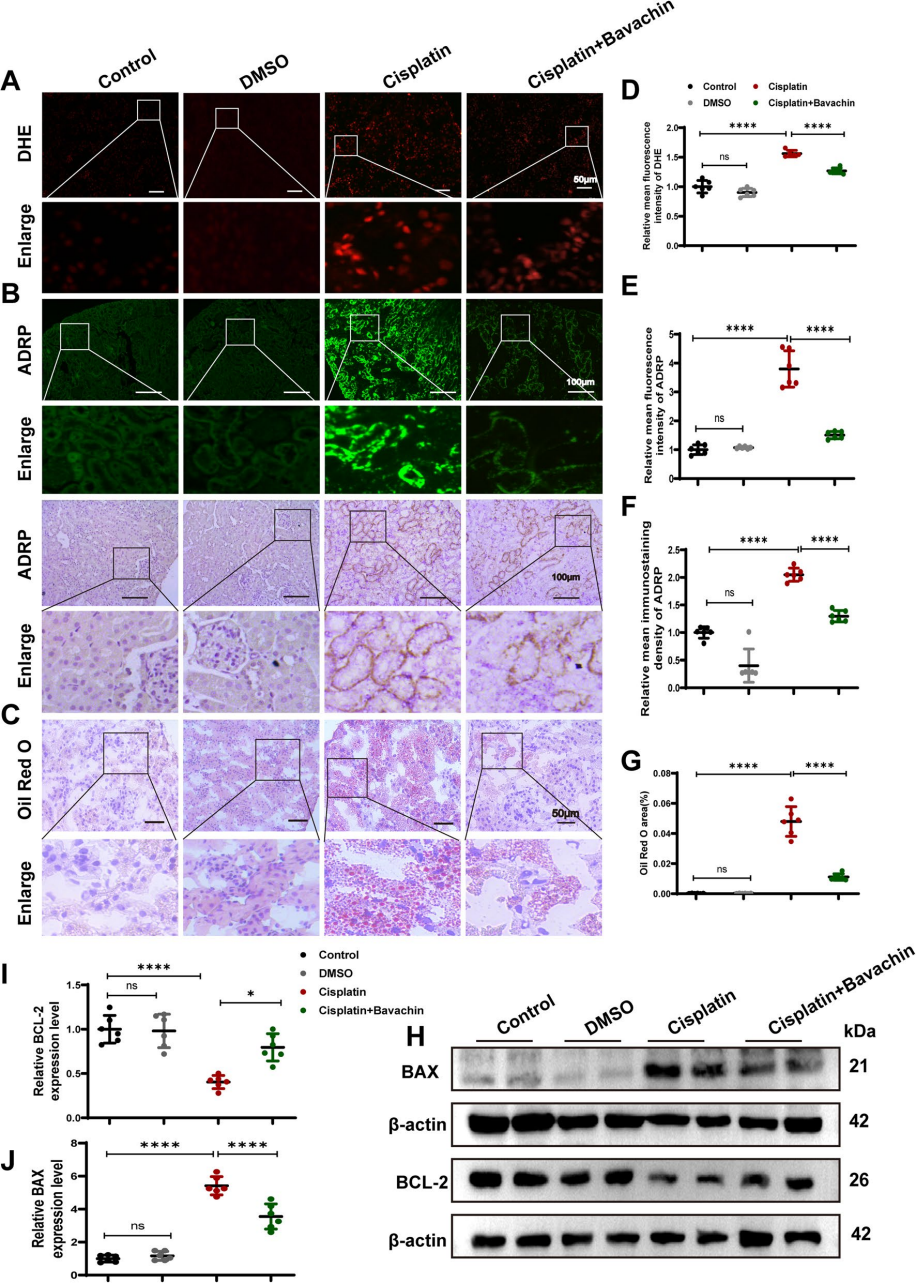
Bavachin attenuates cisplatin-induced renal oxidative stress, lipid accumulation, and cell apoptosis. **A** Representative image of DHE staining. Scale bar = 50 μm.** B** Representative images of immunofluorescence and immunohistochemical staining of ADRP in renal tissues from each group. Scale bar = 100 μm.** C** Representative images of Oil red O staining. Scale bar = 50 μm. **D** Relative mean fluorescence density of DHE. **E** Relative mean fluorescence density of ADRP. **F** Relative immunostaining density of ADRP. Scale bar = 100 μm. **G** Oil Red O area. **H** Representative western blotting analysis of BAX and BCL-2 protein levels in mouse kidneys. **I**, **J** Relative band intensity of BAX and BCL-2. *n* = 6 animals per group. All data were expressed as the mean ± SD (*p < 0.05, ****p < 0.0001, ns: no significant)

As shown in Fig. 2A and D, DHE staining revealed obvious ROS production in the kidneys induced by cisplatin, which can be reduced 20.2% by bavachin. Adipocyte differentiation-related protein (ADRP) is a protein expressed on the surface of lipid droplets in most mammalian cells (Listenberger, et al. 2007). ADRP stimulated lipid accumulation and lipid droplet formation (Imamura, et al. 2002). The expression of ADRP is positively correlated with lipid droplet content, which is a sensitive marker for early adipocyte differentiation (Fan, et al. 2020). Therefore, the lipid deposition was observed by the detection of ADRP by immunohistochemistry and immunofluorescence staining, and it was found that the surface marker ADRP on lipid droplets was significantly upregulated under cisplatin stimulation, whereas was downregulated following intervention with bavachin (Fig. 2B, E and F). In addition, the results of Oil Red O staining similarly demonstrated that bavachin ameliorated cisplatin-induced lipid deposition (76.9%) (Fig. 2C and G). By WB analysis, compared to the control group, treatment of mice with cisplatin notably up-regulated the expressions of BAX and down-regulated the expression levels of BCL-2, whereas these changes were abolished partially after bavachin treatment (Fig. 2H-J).

### Bavachin improves mitochondrial fatty acid β-oxidation by targeting MFN2

**Fig. 3:**
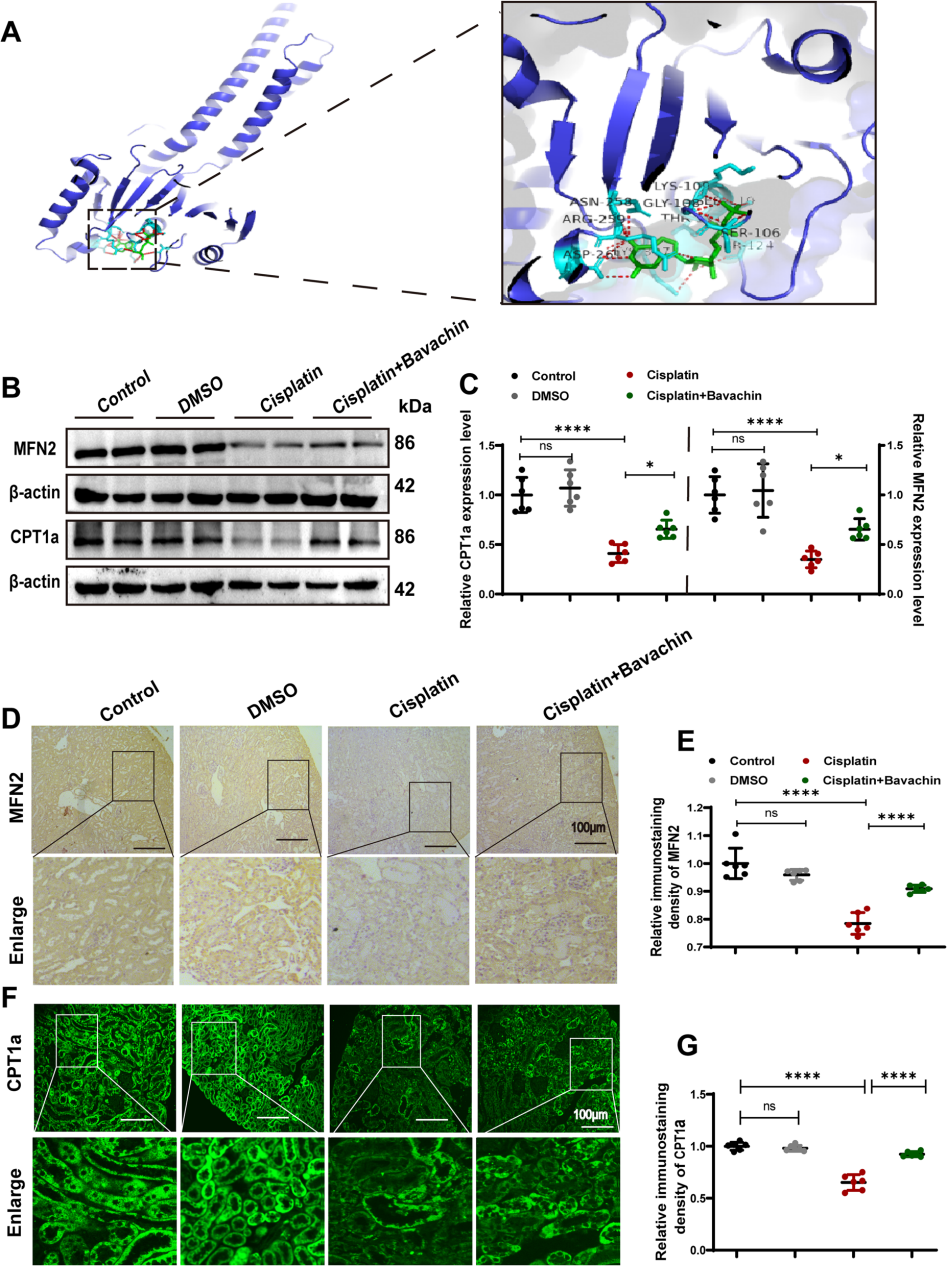
Bavachin improves mitochondrial fatty acid β-oxidation by targeting MFN2. **A** Molecular docking of MFN2 and bavachin. **B** Representative western blotting analysis of MFN2 and CPT1α protein levels in mouse kidneys. **C** Relative band intensity of MFN2 and CPT1α. **D** Representative images of immunohistochemical staining of MFN2 in renal tissues from each group. Scale bar = 100 μm. **E** Relative immunostaining density of MFN2.** F** Representative images of immunofluorescence staining of CPT1α in renal tissues from each group. **G** Relative immunofluorescence density of CPT1α. Scale bar = 100 μm. n = 6 animals per group. All data were expressed as the mean ± SD (*p < 0.05, ****p < 0.0001, ns: no significant)

To investigate potential drug targets for the modulation of lipid metabolism by bavachin, the utilization of the Coremine database revealed that MFN2 emerges as a significant candidate influenced by bavachin and closely associated with lipid metabolism. As illustrated in Fig. 3A, the validation results of molecular docking of bavachin and MFN2 were obtained using AutoDockVina and visualized by PyMOL. Molecular docking revealed that bavachin exhibited a favorable binding energy of −7.28 kcal/mol with the active site of MFN2. Subsequently, WB analysis revealed a downregulation of MFN2 and CPT1α expression in renal tissues of the cisplatin group compared to the control group, which was reversed following treatment with bavachin (Fig. 3B, C). Immunohistochemical staining and immunofluorescence assay further verified that the expression of MFN2 and CPT1α was significantly decreased in renal tissues under cisplatin stimulation, while bavachin treatment effectively increased the expression of MFN2 and CPT1α (Fig. 3D, E, F and G).

### Bavachin attenuates cisplatin-induced oxidative stress, lipid accumulation, and apoptosis in HK-2 cells

**Fig. 4:**
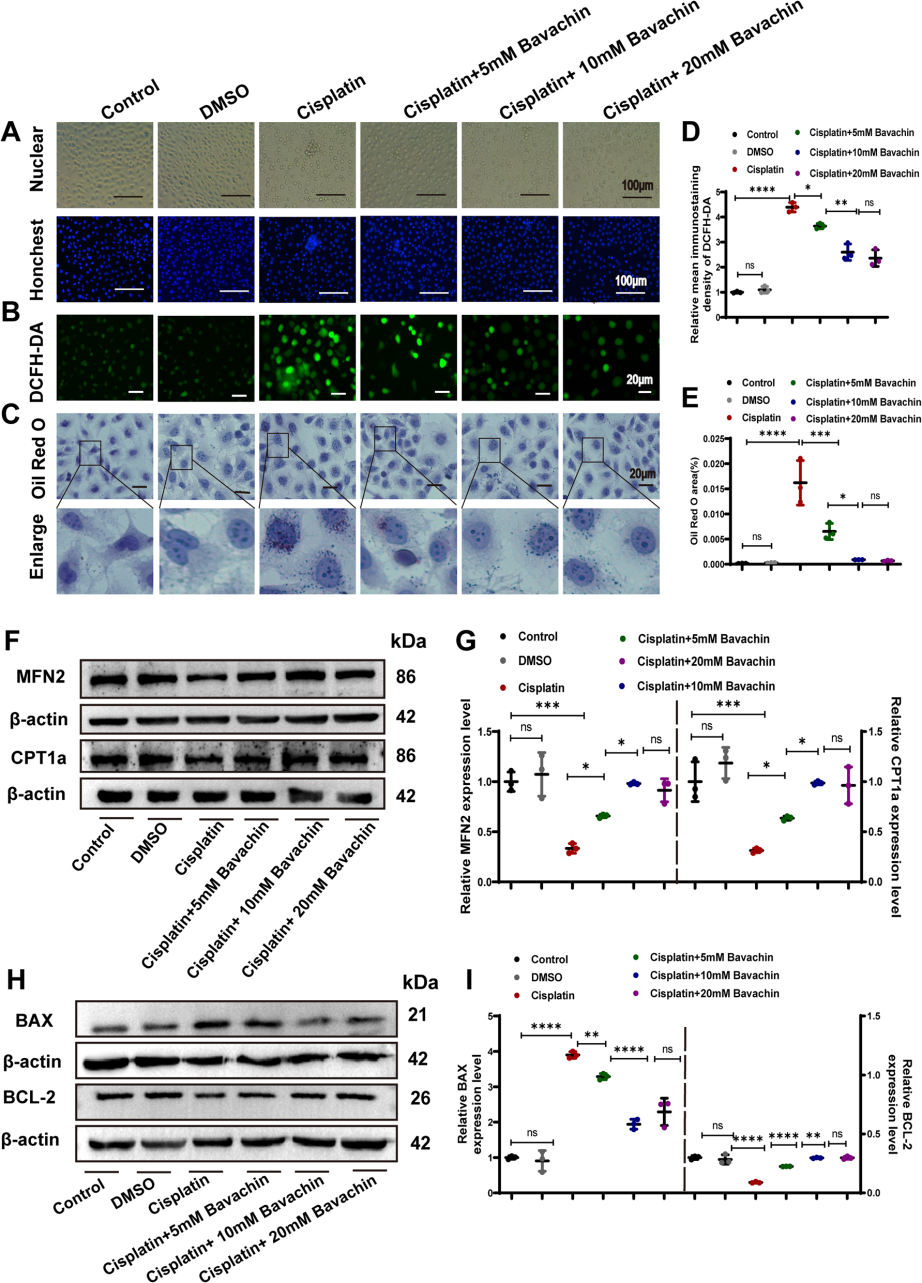
Bavachin attenuates cisplatin-induced oxidative stress, lipid accumulation, and apoptosis in HK-2 cells. **A** Observation of the representative morphology and nucleus of HK-2 cells under a microscope. Scale bar = 100 μm. **B** Representative images of DCFH-DA staining. Scale bar = 20 μm. **C** Representative images of Oil Red O staining. Scale bar = 20 μm. **D** Relative immunofluorescence density of DCFH-DA. **E** Oil Red O area. **F** Representative western blotting image of MFN2 and CPT1α in cisplatin-treated HK-2 cells with or without different concentrations of bavachin. **G** Relative band intensity of MFN2 and CPT1α in cisplatin-treated HK-2 cells with or without different concentrations of bavachin. **H** Representative western blotting analysis of BAX and BCL-2 protein levels in cisplatin-treated HK-2 cells with or without different concentrations of bavachin. **I** Relative band intensity of BAX and BCL-2 in cisplatin-treated HK-2 cells with or without different concentrations of bavachin. *n* = 3. All data were expressed as the mean ± SD (^*^p < 0.05, **p < 0.01, ***p < 0.001, ****p < 0.0001, ns: no significant)

HK-2 cells were exposed to 20 µM cisplatin for 24 h, resulting in a notable increase in apoptosis compared to the control group as indicated by Hoechst 33342 staining. This was characterized by pronounced nuclear shrinkage, condensation, and fragmentation observed under light microscopy. Due to the inconsistent drug concentration of bavachin in different models, we tested the effect of bavachin in the concentration range of 1–50 mM. It was found that 5,10, and 20 mM had a significant concentration-dependent effect, which was also within the effective concentration range of bavachin reported in previous literature (Hung, et al. 2019; Qin, et al. 2021; Wang, et al. 2021). Therefore, the 5,10, and 20 mM bavachin were used for intervention in the following experiments. As shown in Fig. 4A, treatment with 5, 10, and 20 mM bavachin led to improvements in the observed morphological alterations. In addition, DCFH-DA staining revealed an increase in cisplatin-induced ROS production, whereas following treatment with 5, 10, and 20 mM bavachin, the production of ROS gradually decreased (Fig. 4B and D). Furthermore, by Oil Red O staining, cisplatin-induced lipid deposition was enhanced, while significantly alleviated after intervention with 5, 10, and 20 mM bavachin (Fig. 4C and E). WB analysis revealed a decrease in the expression of MFN2 and CPT1α in cisplatin-treated HK-2 cells, which was increased upon bavachin supplementation (Fig. 4F and G). By WB analysis, BCL-2 expression was down-regulated and BAX, cleaved caspase 3 were up-regulated in cisplatin-treated HK-2 cells, which was gradually reversed by bavachin treatment (Fig. 4H, I).

### Bavachin reduces ROS generation and lipid deposition in cisplatin-induced HK-2 cells via an MFN2-dependent mechanism

**Fig. 5:**
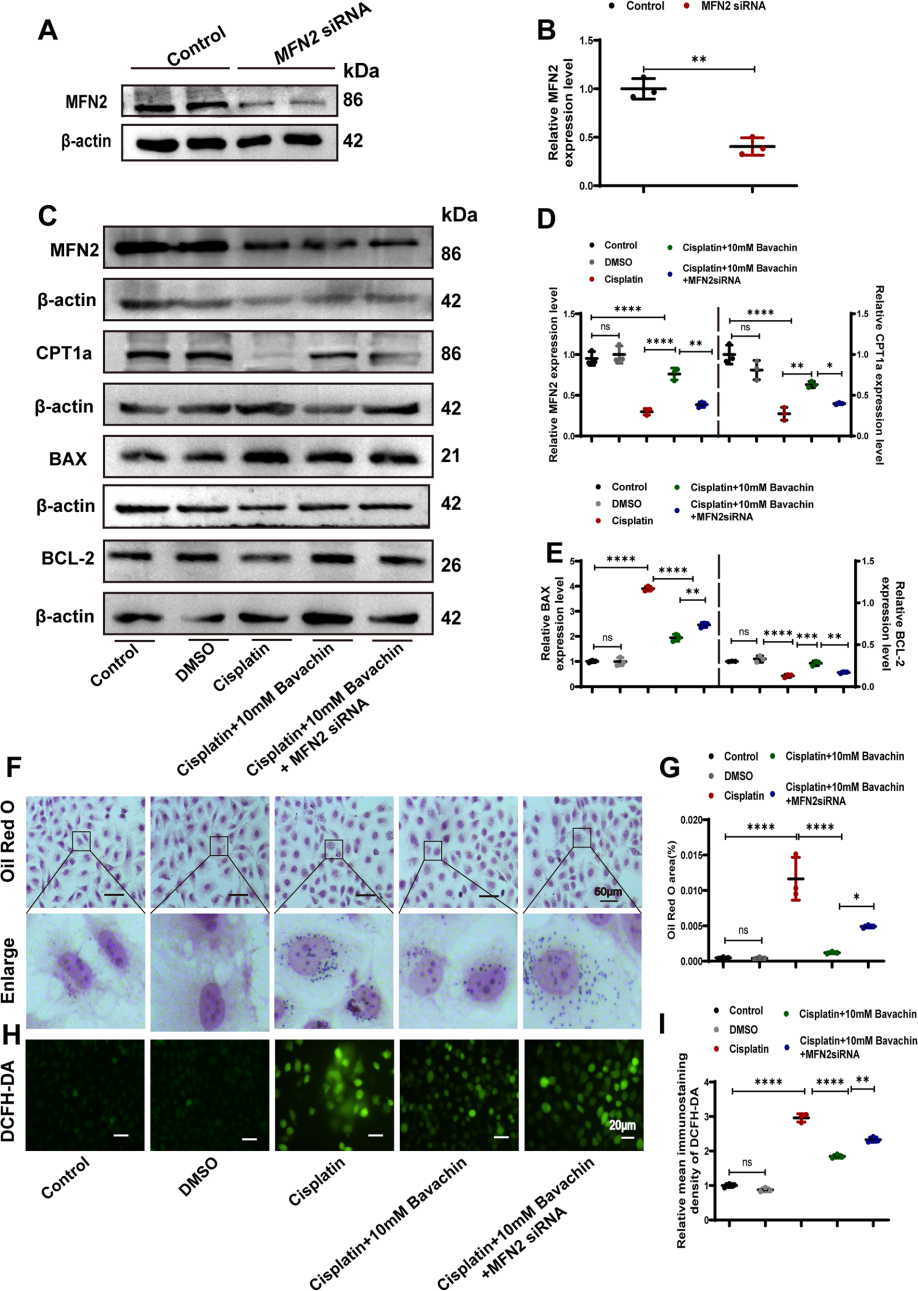
Bavachin reduces ROS generation and lipid deposition in cisplatin-induced HK-2 cells via an MFN2-dependent mechanism. **A** Verification of the knockdown effect of MFN2 siRNA. **B** Relative band intensity of MFN2. **C** Western blot analysis revealed the expression of MFN2, CPT1α, BAX, and BCL-2 in cisplatin-induced HK-2 cells with or without bavachin after siRNA-mediated MFN2 knockdown. **D**-**E** Relative band intensity of MFN2, CPT1α, BAX, and BCL-2. **F** Representative images of Oil Red O staining. Scale bar = 50 μm. **G** Oil Red O area. **H** Representative image of DCFH-DA staining. Scale bar = 20 μm. **I** Relative immunofluorescence density of DCFH-DA. n = 3. All data were expressed as the mean ± SD. (*p < 0.05, **p < 0.01, ***p < 0.001, ****p < 0.0001, *ns*: no significant)

To further investigate whether Bavachin exerted a protective effect against cisplatin-induced nephrotoxicity through MFN2. Firstly, the knockdown effect of MFN2 siRNA was verified (Fig. 5A, B). As shown in Fig. 5C-E, compared with cisplatin-treated HK-2 cells supplemented with bavachin, a significant decrease in the expression of CPT1α and BCL-2 in HK-2 cells could be observed following knockdown of *MFN2* by siRNA. In addition, compared with bavachin supplementation in HK-2 cells treated with cisplatin, the expression of BAX in HK-2 cells significantly increased after knockdown of *MFN2* by siRNA (Fig. 5C-E). Furthermore, Oil Red O staining and DCFH-DA staining were used respectively to detect the lipid deposition and ROS production in HK-2 cells. Compared to cisplatin-treated HK-2 cells supplemented with bavachin, siRNA-mediated *MFN2* knockdown further promoted lipid accumulation and ROS production (Fig. 5F-I).

## Discussion

In the present study, we observed that bavachin significantly attenuated renal tubular cells injury, lipid accumulation, and oxidative stress in the kidneys of cisplatin-induced AKI mice. Furthermore, bavachin increased the expression of MFN2 in cisplatin-treated HK-2 cells and enhanced mitochondrial β-oxidation. These findings suggest that the protective effect of bavachin in cisplatin-induced AKI may involve an MFN2-dependent mechanism.

Bavachin, a natural Chinese medicine extract, is closely related to mitochondrial function and has many pharmacological effects, such as anti-oxidative stress, anti-inflammation, regulation of mitochondrial oxidative phosphorylation, and regulation of insulin signal. Currently, there are limited clinical protective agents for AKI. Previous studies have shown that nebivolol (Abd-Eldayem, et al. 2025), Smilax glabra roxb. (Zhao, et al. 2025), and papaverine (Abass, et al. 2024) have a certain protective effect on AKI. However, in contrast to this class of drugs, bavachin mainly has the property of regulating lipid metabolism (Wei, et al. 2023). Recent study has demonstrated that bavachin improves liver steatosis and obesity by inhibiting the expression of genes associated with high glucose or high fat diet-induced fatty acid synthesis and increased lipid droplet numbers (Wei, et al. 2023). However, little is known about the long-term renal protective effects of bavachin as a natural product in AKI or its potential side effects. At present, the preclinical pharmacokinetic data of the drug are still incomplete, its bioavailability and drug interactions need to be further evaluated, and the potential risks of long-term exposure have not been systematically evaluated. Notably, Park et al. found that oxidative stress and enhanced mitochondrial function were improved in db/db mice by oral administration of bavachin (10 mg/kg) via intubation for 6 weeks, and glomerular area and renal collagen deposition were significantly reduced (Park, et al. 2023). This suggests that bavachin may have unique value in blocking the transition from AKI to CKD. However, the long-term efficacy of bavachin in the prevention and treatment of AKI to CKD remains to be further investigation.

Renal tubular epithelial cells are energy-intensive cells with fatty acid metabolism as the main energy source, and lipid metabolism deficiency is a key mediator in the progression from AKI to renal fibrosis (Kang, et al. 2015) (Schaub, et al. 2021; Scholz, et al. 2021; Tammaro, et al. 2020). Previous studies have shown that increased fatty acid metabolism and upregulation of fatty acid oxidation (FAO)-related genes have a protective effect on cisplatin-induced AKI. Xu et al. found that the FXR/PPARγ signaling pathway interferes with cisplatin-induced lipid deposition and kidney injury by improving FAO (Xu, et al. 2022). Additionally, up-regulation of PGC-1α/PPARα/CPT1Α signaling also has a protective effect on cisplatin-induced AKI (Wang, et al. 2024). In this study, we found that bavachin significantly reduced cisplatin-induced nephrotoxicity and upregulated MFN2/CPT1a expression. Previous studies have shown that CPT1a overexpression can prevent mitochondrial dysfunction and restore FAO and ATP levels in the Unilateral Ureteral Obstruction (UUO) model (Miguel, et al. 2021). CPT1Α not only promotes lipid consumption through the fatty acid metabolic pathway, thereby reducing lipotoxicity, but also anchoring Bcl2 to the mitochondrial membrane, thereby preventing cytochrome C release and inhibiting the mitochondrial apoptotic process (Xie, et al. 2024) In CPT1a-null lung cancer cells, reduced NADPH and reduced glutathione were observed, while free fatty acid levels were increased. Mechanistically, CPT1a interacted with c-Myc to block the ubiquitination and degradation of c-Myc by FBXW7, activate NRF2/GPX4 pathway, reduce the accumulation of phospholipid polyunsaturated fatty acids, and inhibit ferroptosis (Ma, et al. 2024). These results suggest that bavachin can reduce lipid metabolic fence and attenuate cisplatin -induced lipid kidney injury by up-regulating MFN2/CPT1a and improving mitochondrial FAO.

What needs to be explored next is how bavachin regulates the oxidative metabolism of fatty acids. Mitochondrial fatty acid metabolism is regulated by a variety of proteins, and MFN2 plays an important role in mitochondrial oxidation capacity in addition to controlling the morphology of the mitochondrial network. In the study by Mingrone et al., it was mentioned that a decrease in *MFN2* mRNA levels in obese patients is associated with a reduced glucose oxidation capacity (Mingrone, et al. 2005). Subsequently, an increasing number of studies have indicated a close relationship between MFN2 and mitochondrial energy metabolism. The deficiency of *Mfn1* and *Mfn2* led to abnormal mitochondrial morphology and lipid metabolism disorder in AEC2 cells, with a significant decrease in cholesterol and specific glycerophospholipids, affecting the normal synthesis of pulmonary surfactants, thereby promoting the progression of pulmonary fibrosis (Chung, et al. 2019). More importantly, MFN2 was found to promote mitochondrial FAs β-oxidation in Pelteobagrus fulvidraco hepatocytes through its GTPase domain interaction with CPT1α (Song, et al. 2024). Furthermore, in clear cell renal cell carcinoma (ccRCC), MFN2 can inhibit the progression of ccRCC by blocking cellular lipid metabolism and reducing lipid droplet accumulation (Cai, et al. 2024). In our study, we found that MFN2 is one of the target proteins of the bavachin, which can upregulate the protein expression level of MFN2. This suggests that MFN2 may be a key target molecule for bavachin to protect against cisplatin-induced nephrotoxicity. In addition, MFN2 expression is regulated by AMPK to enhance mitochondrial fusion and reduce ROS production in a mouse model of IRI injury (Zhang, et al. 2023). In adipocytes, bavachin not only activated AMPK signaling pathway but also activated the transcriptional activity of PPARγ, promoting its expression, and up-regulating the expression of adiponectin and GLUT4, thereby improving insulin sensitivity and glucose uptake (Lee, et al. 2016). However, in hepatocytes, bavachin can induce ER stress through AMPK/mTORC1 signaling (Yang, et al. 2021)Therefore, in addition to this study suggesting that bavachin regulates mitochondrial β-oxidation through MFN2-dependent mechanism, whether bavachin protects AKI by regulating mitochondrial metabolic network, especially the above molecules, remains to be further proved in the future.

To further investigate the role of MFN2 in the reno-protective effect of bavachin in cisplatin-induced AKI, our experiments observed that knockdown *MFN2* in HK-2 cells could prevent bavachin from improving lipid accumulation, oxidative stress, and cell apoptosis induced by cisplatin. The above data suggest that MFN2 is involved in the reno-protective effect of bavachin in cisplatin-induced AKI. In addition, Zhang et al. found that *Klf9* knockdown resulted in mitochondrial disorder, impairing mitochondrial respiratory and contractile function in cardiomyocytes, which could be rescued by adeno-associated virus-mediated *Mfn2* (Zhang, et al. 2024). In addition, MFN2-mediated mitochondrial fusion plays an important role in the critical period of synaptic plasticity in adult newborn neurons. Conditional knockout of *Mfn1* or *Mfn2* resulted in fragmentation of mitochondrial network, reduced dendritic complexity, and decreased survival of neurons (Kochan, et al. 2024), suggesting that MFN2, as an important protein regulating mitochondrial network and metabolism, may also play an important role in other diseases related to abnormal mitochondrial dynamics.

In this study, we demonstrated that bavachin protected cisplatin-induced HK-2 cells lipotoxicity by regulating MFN2/CPT1a expression and improving mitochondrial β oxidation. Here, cisplatin was used to induce AKI, as it has been verified to have a stable model of lipid metabolism disorder (Xu, et al. 2022). However, the nephrotoxicity of cisplatin in vivo is not only related to direct cell injury, but also closely related to immune cells infiltration, inflammatory factor cascade and hemodynamic disorder (Peres and da Cunha 2013; Fang, et al. 2021; Volovat, et al. 2023), and these systemic factors are difficult to reproduce in vitro models. Second, in vitro experiments typically employ fixed concentrations and short drug treatment times, while the toxic effects of cisplatin in clinical use may be dynamically variable due to metabolic differences, dosing cycle, and individual factors. In addition, HK-2 cells lack the multi-cellular synergies of in vivo nephron, such as endothelial cells, interstitial cells, etc., which may underestimate or overestimate the role of MFN2 in overall organ functional repair.

### Limitation

This study is a small sample size animal and cell experiment, mainly to observe the lipotoxicity-protective effect of bavachin on cisplatin-induced AKI, and the long-term observation of AKI efficacy on CKD is lacking, which is worthy of further study. In addition, in future study, we will further focus on the effect of bavachin on cellular oxidative metabolism to provide experimental research for clinical cisplatin combined with bavachin treatment of tumors.

## Conclusion

Currently, the lack of specific therapies for renal tubule mitochondrial metabolic disorders and the dose-limiting nephrotoxicity of cisplatin severely limit the efficacy of tumor therapy. In this study, bavachin precisely regulated mitochondrial β-oxidation through MFN2-CPT1α axis, significantly reduced cisplatin-induced lipid accumulation, and directly improved AKI metabolic arrest by promoting FAO through activation of CPT1α.

## Supplementary Information

Supplementary Material 1.

Supplementary Material 2.

## Data Availability

The raw data supporting the conclusions of this article will be made available by the authors, without undue reservation.

## Abbreviations

AKI: Acute kidney injury
ROS: Reactive oxygen species
MFN2: Mitofusin 2
FAO: Fatty acid oxidation
H&E staining: Hematoxylin and eosin staining
BUN: Blood urea nitrogen
SCr: Serum creatinine
CHOL: Cholesterol
NAFLD: Nonalcoholic fatty liver disease
DAPI: 4,6-Diamidino-2-phenylindole
DHE: Dihydroethidium
