# Correction: Bavachin ameliorates cisplatin-induced nephrotoxicity by enhancing mitochondrial β-oxidation and lipid metabolism through MFN2

**DOI:** 10.1186/s10020-026-01443-2

**Published:** 2026-02-28

**Authors:** Shilu Luo, Ming Yang, Na Jiang, Chenrui Li, Yan Liu, Lin Sun

**Affiliations:** 1 Department of Nephrology, the Second Xiangya Hospital, Central South University, No. 139 Renmin Middle Road, Changsha, Hunan 410011 China; 2 Hunan Key Laboratory of Kidney Disease and Blood Purification, No. 139 Renmin Middle Road, Changsha, Hunan 410011 China

**Correction: Molecular Medicine 31, 234 (2025)**

**https://doi.org/10.1186/s10020-025-01283-6**

In the originally published article [1], the in vivo dosage of Bavachin was inadvertently omitted from the "*Animals model*" section under "*Materials and methods*".

The corrected paragraph should read as follows:

Animals model

6-to-8-week male C57BL/6 mice (body weight range: 21–25 g) purchased from Hunan SJA Laboratory Animal Co.Ltd. All mice were placed in a quiet environment (temperature: 21–24 °C; Humidity: 50 ± 5%) and had free access to water and standard chow. All experimental mice were randomly divided into control, DMSO, cisplatin, and cisplatin + Bavachin group. The male C57BL/6 mice in the cisplatin group were injected with cisplatin (20 mg/kg, cat # 479306, Sigma, United States) intraperitoneally and the control group was given the same volume of saline. Mice in the cisplatin + Bavachin group were administered Bavachin (20 mg/kg) by single intraperitoneal injection, as previously described [Antioxidants (Basel). 2022 Oct 24;11(11):2096. doi: 10.3390/antiox11112096] and [Acta Pharmacol Sin. 2023 Jul;44(7):1416-1428. doi: 10.1038/s41401-023-01056-z]. Bavachin (MedChemExpress, HY-N0233) was dissolved in 10% dimethyl sulfoxide and 90% saline mixed with sulfobutylether-β-cyclodextrin (MedChemExpress, HY-17031). All mice were euthanized with pentobarbital at 72 h. All animal experiments were performed by the protocols approved by the Institutional Animal Care and Use Committee of the Second Xiangya Hospital of Central South University.

The original article has been corrected.
