# Supplementary figures and images for: Bavachin ameliorates cisplatin-induced nephrotoxicity by enhancing mitochondrial β-oxidation and lipid metabolism through MFN2
Source: Mol Med. 2025 Jun 11;31:234. doi: 10.1186/s10020-025-01283-6 (PMC12160371; doi:10.1186/s10020-025-01283-6)

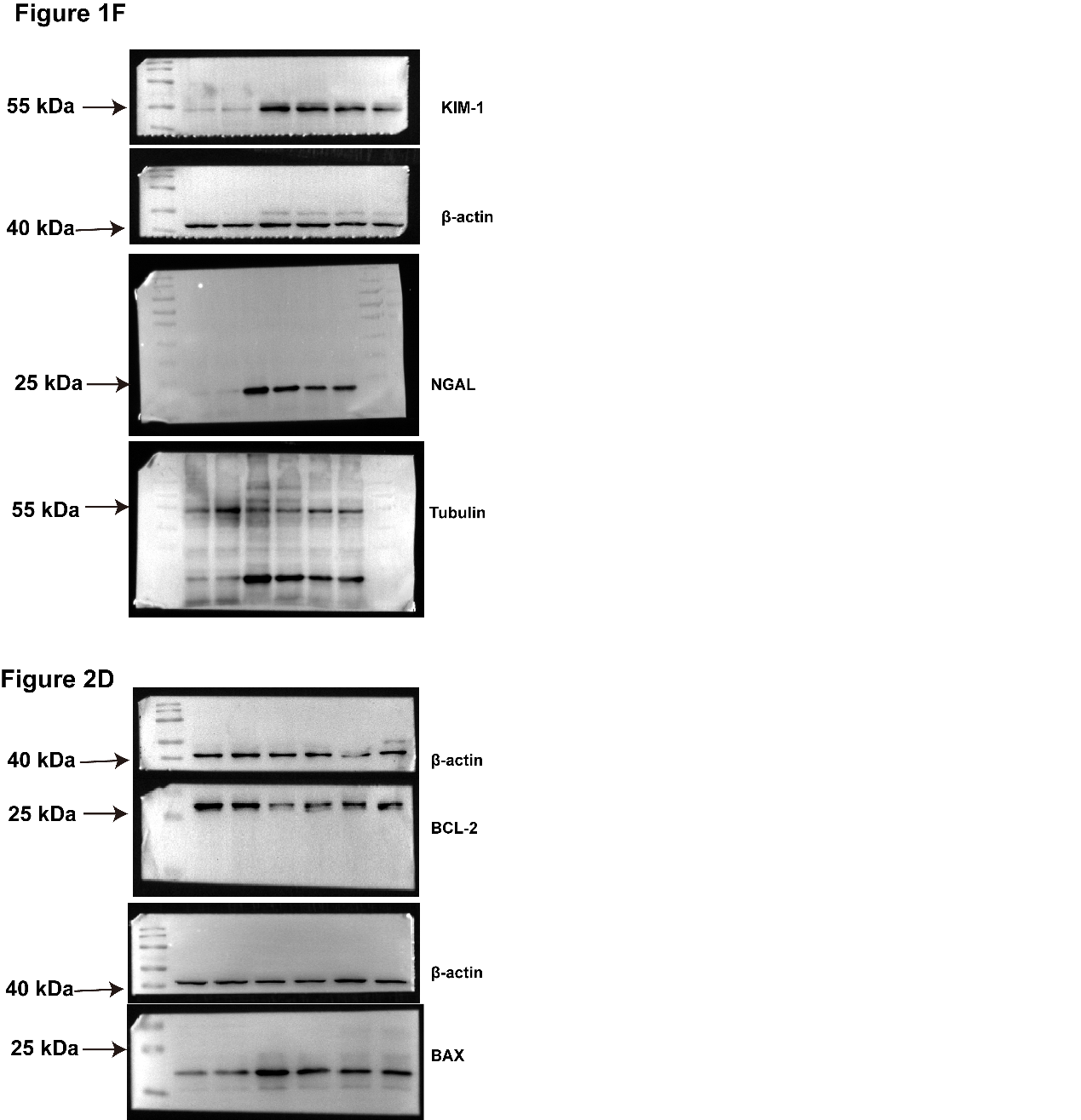

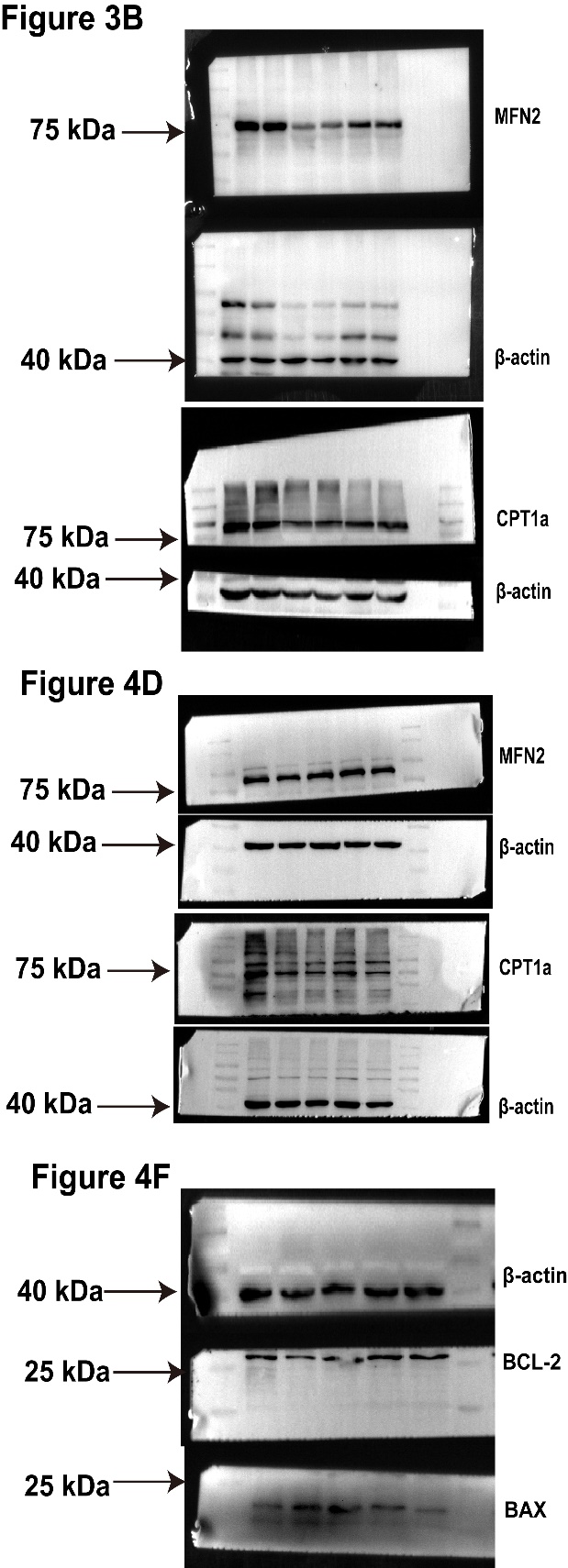

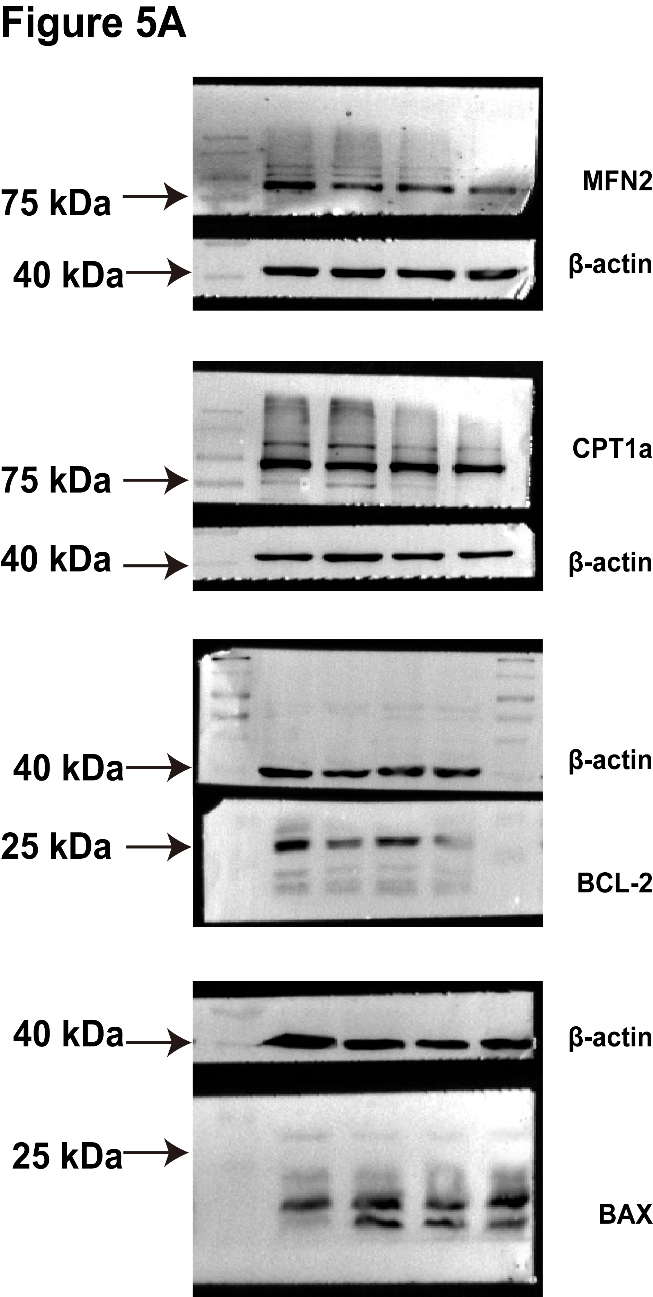

Supplement: Supplementary file 1 — Supplementary Material 1. [file 10020_2025_1283_MOESM1_ESM.docx]

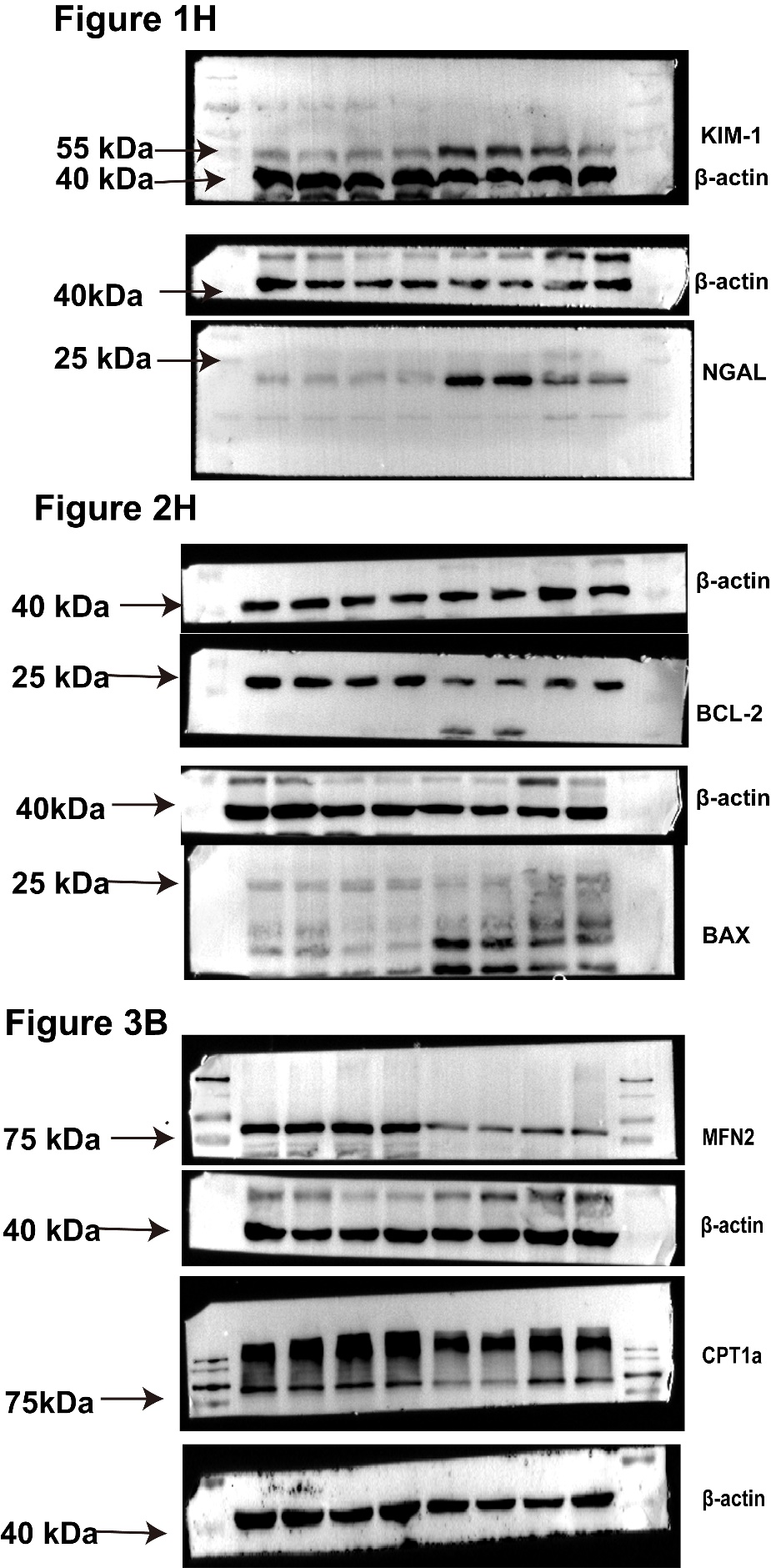

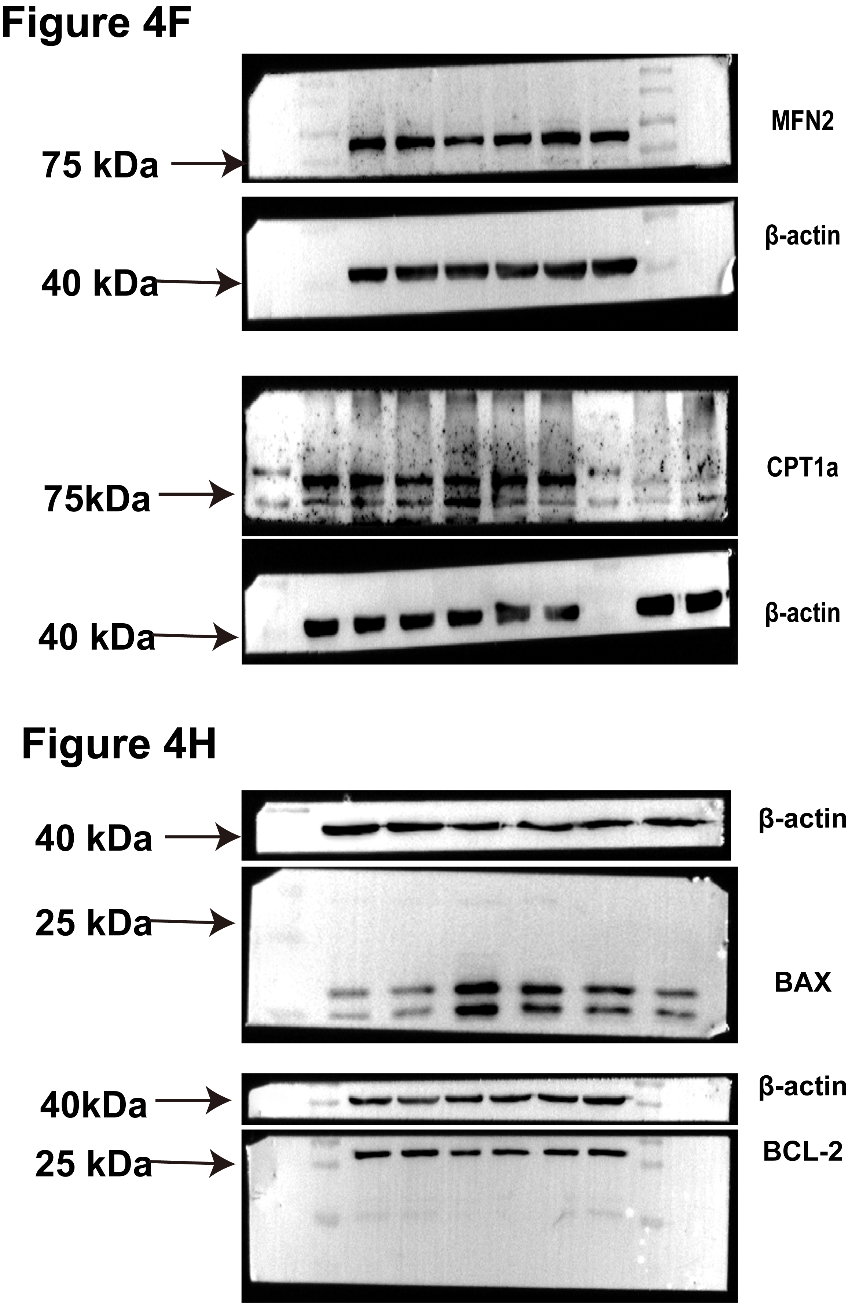


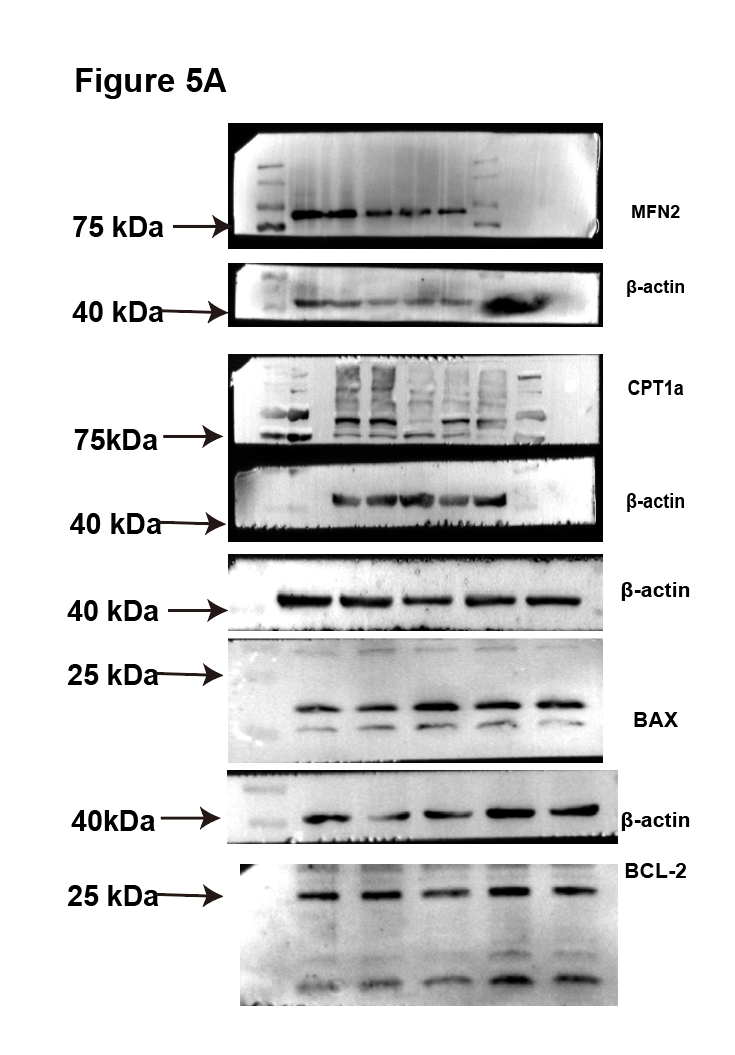

Supplement: Supplementary file 2 — Supplementary Material 2. [file 10020_2025_1283_MOESM2_ESM.docx]
